# Internal rotation strength recovers fully after the open Latarjet via a subscapularis‐split: A systematic review and meta‐analysis

**DOI:** 10.1002/jeo2.70882

**Published:** 2026-08-06

**Authors:** Willem Geuskens, Arjun Mehra, Eoghan Hurley, Hannan Mullett

**Affiliations:** ^1^ Royal College of Surgeons in Ireland Dublin Ireland; ^2^ UPMC Sports Surgery Clinic Dublin Ireland; ^3^ Department of Orthopedic Surgery University Hospitals Leuven Leuven Belgium; ^4^ University of Limerick Limerick Ireland; ^5^ Department of Orthopaedic Surgery Duke University Durham North Carolina USA

**Keywords:** isokinetic strength, Latarjet, return to sports, shoulder instability, shoulder rotational strength

## Abstract

**Purpose:**

To systematically evaluate postoperative internal rotation (IR) and external rotation (ER) strength recovery following the open Latarjet (OL) procedure performed through a subscapularis‐split approach using isokinetic dynamometry.

**Methods:**

A systematic search of PubMed, Embase, and the Cochrane Library following PRISMA guidelines was performed to identify clinical studies reporting postoperative shoulder rotational strength after OL. Extracted outcomes included IR and ER peak torque and ER/IR ratios. A random‐effects meta‐analysis was conducted for studies reporting peak torque at 60°/s.

**Results:**

Eleven studies encompassing 274 patients met inclusion criteria. Across studies, rotational strength recovery followed a consistent temporal pattern. Early (<6 months) deficits were substantial for IR (18%–42%) and ER (8%–30%). Between 6 and 12 months, strength improved but did not fully normalise. At ≥18 months, IR strength typically recovered, whereas small but statistically significant ER deficits persisted (4%–15%). Meta‐analysis demonstrated pooled deficits of 17.0% for ER (95% confidence interval [CI], 12.1–21.9) and 15.1% for IR (95% CI, 9.0–21.2), with significantly smaller deficits at late compared with early follow‐up for both IR and ER strength. ER/IR ratios showed no overall asymmetry; however, subgroup analysis revealed a shift from early IR‐dominant weakness to late ER‐dominant weakness.

**Conclusions:**

Shoulder rotational strength recovery after the OL procedure through a subscapularis‐split approach follows a predictable temporal pattern, with early internal rotation deficits resolving by 6–12 months and small but persistent external rotation deficits at long‐term follow‐up. These findings indicate that the split of the subscapularis muscle does not compromise IR strength at long‐term follow‐up, supporting its safety for young and active patients.

**Level of Evidence:**

Level III.

**Abbreviations**
ER: external rotation
IR: internal rotation
JBI: Joanna Briggs Institute
LSI: Limb Symmetry Index
NHMRC: National Health and Medical Research Council
OL: open Latarjet procedure
PRISMA: Preferred Reporting Items for Systematic Reviews and Meta‐Analyses
PROMs: Patient‐Reported Outcome Measures
RoB: risk of bias
RTS: return‐to‐sport
SD: standard deviation

## INTRODUCTION

In recent years, the open Latarjet (OL) procedure has become increasingly popular in the management of anterior shoulder instability due to its excellent outcomes, including high return to sports (RTS) rates and low recurrent instability rates [15, 19]. Although the OL procedure was historically reserved for revision cases or patients with significant bone loss, it is now frequently used as a primary stabilisation technique, particularly for athletes and active individuals who require robust shoulder stability for high‐demand activities [12].

However, the OL is considered a ‘non‐anatomic’ procedure because it involves the transfer of the coracoid process through a horizontal split of the subscapularis muscle, thereby altering the working length of the conjoint tendon. This modification, together with the release of the pectoralis minor muscle, results in a more posteriorly tilted and protracted scapula relative to the thorax [3, 11].

Although the biomechanical implications of the OL procedure have been studied to some extent, the functional implications of this ‘non‐anatomic’ procedure remain poorly understood. In particular, postoperative shoulder rotational strength recovery—an important determinant of functional recovery and return to sport—has not been systematically characterised, and the strength recovery pattern of internal and external rotation has not been clearly defined.

Therefore, the purpose of this systematic review was to evaluate postoperative shoulder rotational strength recovery following the OL procedure, quantified as peak internal and external rotation torque using isokinetic dynamometry.

## METHODS

This systematic review was conducted in accordance with the Preferred Reporting Items for Systematic Reviews and Meta‐Analyses (PRISMA) guidelines [23].

### Databases and search engines

A search of the databases PubMed, Cochrane and Embase was conducted in April 2026 with the purpose of collecting clinical studies that investigated the postoperative shoulder rotational strength after the OL procedure.

### Applied search limits

The search was limited to English language articles published before April 2026.

### Eligibility criteria

#### Inclusion criteria

Clinical studies that included cohorts of patients with shoulder instability treated with the OL procedure and assessed postoperative shoulder rotational strength using an isokinetic dynamometer were included. Postoperative rotational strength was required to be compared with one of the following: preoperative rotational strength of the injured limb, the uninjured limb, or an uninjured control group. Included study designs comprised cross‐sectional case‐control studies, randomised controlled trials, prospective or retrospective cohort studies, and case series.

#### Exclusion criteria

Papers written in a language other than English were excluded. Studies investigating cohorts with patients older than 50 years, heterogeneous cohorts that included other stabilisation procedures (e.g., arthroscopic Bankart repair or open Bankart repair), patients experiencing shoulder instability due to epileptic seizures, or OL procedures performed via a subscapularis tenotomy were excluded. Studies assessing shoulder rotational strength using tools other than an isokinetic dynamometer and case reports were also excluded.

### Search terms

A systematic literature search was performed in PubMed, Cochrane and Embase to identify clinical studies reporting postoperative shoulder rotational strength after the OL procedure using an isokinetic dynamometer. The search was performed in April 2026. The search was limited to articles published in English before 1 April 2026. Search terms were developed according to the PICO framework and combined terms related to shoulder rotational strength, isokinetic testing, and the Latarjet procedure. The complete search syntax used for each database is provided in Supporting Information: Appendix S1. Citation mapping, grey literature searches, and additional reference‐list screening were not performed.

### Study selection

After removing duplicates, two independent reviewers screened the titles and abstracts to identify studies that met the inclusion criteria. A detailed full‐text analysis was then conducted to determine which articles to include in the systematic review. Disagreements between the reviewers were resolved through discussion or by consulting a third author.

### Data extraction

Data extraction was performed independently by two reviewers using a predefined data template. Extracted publication characteristics included the first author, year of publication, journal, and PubMed Unique Identifier (PMID).

Study characteristics included study design, sample size, follow‐up duration, and whether the OL procedure was performed as a primary or revision stabilisation procedure. Patient characteristics consisted of age, sex, dominant‐limb involvement, and sports participation.

Details of isokinetic strength testing were also extracted, including the warm‐up protocol, testing position, number of repetitions, angular velocity, and dynamometer protocol. Strength outcomes of interest included peak torque for internal rotation (IR) and external rotation (ER), the ER/IR ratio, whether strength was reported as absolute torque or relative torque, and comparison (contralateral uninjured limb, an uninjured control group, or preoperative strength of the injured limb).

### Quality assessment

Risk of bias and quality appraisal in each study was assed using the Joanna Briggs Institute (JBI) critical appraisal tool. The level of evidence of each study was graded according to the Australian National Health and Medical Research Council (NHMRC) criteria [13].

### Data analysis

A quantitative synthesis was performed for studies that reported on isokinetic rotational strength recovery at an angular velocity of 60°/s. Outcomes included side‐to‐side (1) external rotation (ER) strength deficits, (2) internal rotation (IR) strength deficits, and (3) ER/IR ratio differences between the injured and uninjured limbs. These deficits and differences were expressed as percentages of the uninjured side. Only studies reporting mean and standard deviation (SD) of peak torque values or directly providing percent deficits relative to the contralateral shoulder were included in the quantitative synthesis. When not explicitly reported, deficits and SDs were calculated from raw values of the injured and uninjured limbs using standard methods for continuous data [14].

A random‐effects model (DerSimonian–Laird estimator) was applied to account for expected between‐study variability. Results are reported as pooled mean percentage differences with 95% confidence intervals (CIs) and 95% prediction intervals (PIs) to indicate the range in which true effects are likely to occur in future comparable populations.

Heterogeneity was quantified using Cochran's Q, *I* ^2^, and τ^2^ statistics, with *I* ^2^ values above 75% considered indicative of substantial heterogeneity. Potential moderators were explored using subgroup analysis, comparing outcomes between early (< 6 months) and late (≥ 12 months) postoperative testing intervals. When multiple postoperative time points were available from the same cohort, each time point was treated as an independent stratum representing a distinct recovery interval, consistent with longitudinal meta‐analytic methodology. The significance of subgroup differences was assessed using the omnibus Qm statistic. Forest plots were visually inspected to assess effect consistency and precision. Assessment of publication bias using funnel plots was planned if ≥10 studies contributed to a given meta‐analysis. All *p*‐values were two‐tailed, with *p* < 0.05 considered statistically significant.

## RESULTS

### Selection of the studies

**Figure 1:**
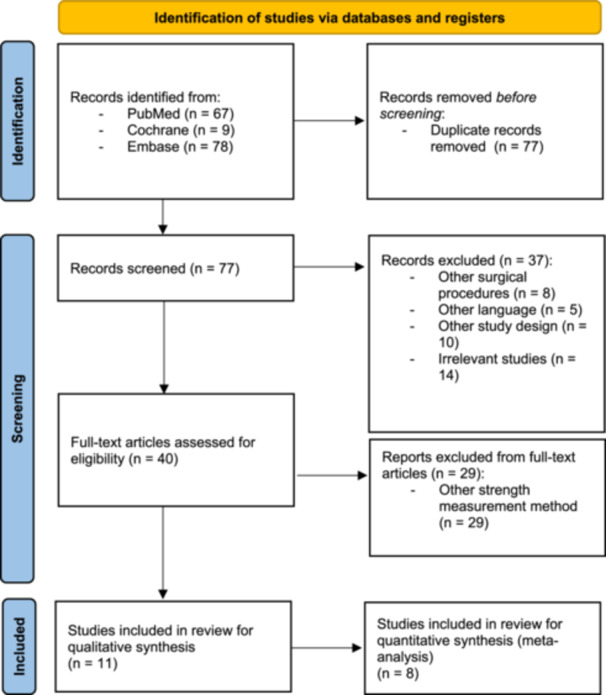
PRISMA flow diagram of study inclusion. PRISMA, Preferred Reporting Items for Systematic Reviews and Meta‐Analyses. This work is licensed under CC BY 4.0. To view a copy of this license, visit https://creativecommons.org/licenses/by/4.0/. *Source*: Page et al. [23].

153 articles were identified through the initial database searches. After removing duplicates, title screening and full‐text article assessment for eligibility, 11 studies were eventually included in this study (Figure 1).

### Quality assessment

Using the JBI tools, three studies had a low RoB, seven a moderate RoB, and one a high RoB (Table 1). Prospective designs achieved the highest JBI scores (8/10 or 9/11) [8, 9, 21]. The remaining studies had a moderate RoB due to retrospective designs, lack of control groups, small sample sizes, or limited adjustment for confounding variables. One early study demonstrated high RoB because of incomplete methodological reporting [26]. According to the NHMRC criteria, the majority of studies were Level IV case series, with three Level III‐2 comparative cohorts and one Level II prospective cohort.

**Table 1:** Study characteristics.

Study ID	First author	Year	Design (for JBI*)	JBI tool	JBI score	Risk of bias	NHMRC level
1	Ribeiro	2021	Analytical cross‐sectional study	Cross‐sectional	5/8	Moderate	IV
2	Caubère	2017	Retrospective comparative cohort study	Cohort	8/11	Moderate–Low	III‐2
3	Edouard	2012	Prospective cohort study	Cohort	7/11	Moderate	III‐2
4	Edouard	2010	Prospective case series	Case Series	8/10	Low	IV
5	Ernstbrunner	2022	Retrospective case series	Case Series	7/10	Moderate	IV
6	Malavolta	2020	Prospective case series	Case Series	7/10	Moderate	IV
7	Moussa	2024	Prospective cohort study	Cohort	9/11	Low	II
8	Reddy	2024	Retrospective case series	Case Series	6/10	Moderate	IV
9	Ersen	2018	Retrospective comparative cohort study	Cohort	8/11	Moderate–Low	III‐2
10	Edouard	2012	Prospective case series	Case Series	8/10	Low	IV
11	Wredmark	1992	Retrospective case series	Case Series	5/10	Moderate–High	IV

Abbreviations: JBI, Joanna Briggs Institute; NHMRC, National Health and Medical Research Council; RTS, Return to sports.

### Results of the clinical trials

#### Study Selection and demographics

The three studies by Edouard et al. investigated the same patient cohort at different postoperative time points and were therefore not treated as independent studies (Table 2). This resulted in a total of nine unique patient cohorts and 274 patients across the eleven included studies [7, 8, 9]. All the studies were published between 1992 and 2024, with a steady increase in the number of studies in the past decade.

**Table 2:** Patient demographic.

Study ID	Sample size	Mean age (year)	Gender (M/F)	BMI	Dominant limb affected (%)	Participation in sports (%)	Average follow‐up (months)	First time dislocation vs. recurrent instability	Number of dislocations prior to surgery	Primary (P) or revision (R) surgery or combination (C)
1	18	27	N/A	N/A	N/A	100%	N/A	N/A	N/A	Primary
2	20	25	100%	24.3	60%	100%	12	Recurrent	3	Primary
3	20	27	100%	22.78	40%	85%	21	Recurrent	4	Primary
4	20	27	100%	22.78	40%	85%	21	Recurrent	4	Primary
5	42	26	90%	N/A	62%	95%	101	Recurrent	N/A	Primary
6	20	37	95%	N/A	65%	100%	36	Recurrent	26	Primary
7	72	27	83%	24	N/A	100%	6	Recurrent	3.6	Primary
8	10	20	80%	25	N/A	100%	5	Recurrent	N/A	Combination
9	28	34	85.70%	N/A	64.30%	N/A	19	Recurrent	12.6	Combination
10	20	27	100%	22.78	40%	85%	3	Recurrent	4	Primary
11	44	30	79.50%	N/A	90%	N/A	72	Recurrent	>5	Combination

Abbreviations: BMI, body mass index; N/A, not applicable.

Most patients were young, physically active males who participated in contact and collision or overhead sports, such as rugby, football, judo, or handball. The mean age reported in the included cohorts ranged from 20 to 34 years and 80% to 100% of the cohorts consisted of male patients. Sports participation ranged from 85% to 100% and the dominant limb was affected in 40%–90% of the cases. Nine out of the eleven studies reported on the recurrency of shoulder instability prior to surgery. In these cohorts, all the included patients suffered from recurrent anterior shoulder instability. The mean number of dislocations prior to surgery ranged from 4 to 26. Eight of the eleven studies investigated the outcomes of the OL procedure as a primary stabilisation procedure. The three other studies also included patients who underwent previous arthroscopic stabilisation. None of the studies included patients that underwent a bone block procedure prior to the OL procedure.

#### Surgical procedure

All stabilisation procedures were performed using an OL technique through a subscapularis split. Four studies reported additional capsulolabral repair or plication concurrent with bone block fixation.

#### Isokinetic testing protocols

Isokinetic strength testing was consistently performed using either a Cybex® or Con‐Trex® isokinetic dynamometers, assessing concentric IR and ER peak torque at different angular velocities (Table 3). The angular velocities tested ranged between 30°/s and 240°/s; 60°/s was most common (*n* = 9), followed by 180°/s (*n* = 8) and 240°/s (*n* = 4). Testing was performed either seated (*n* = 10) or standing (*n* = 1) with the arm adducted at 0° (*n* = 5), abducted at 45° (*n* = 5) or abducted at 90° (*n* = 1). Torque was expressed as Nm/kg in five studies, Nm in three studies, and as limb symmetry index (LSI) in the remaining three studies. Ten studies compared the isokinetic strength of the injured limb with the uninjured limb, two studies with the preoperative strength of the injured limb and one study included a healthy control group and a conservatively managed control group for comparison.

**Table 3:** Isokinetic testing protocols.

Study ID	Postition	Arm position (° of abduction)	Warm‐up protocol	Number of repetitions	speed (°/s)	Peak torque measurement	Outcome measurement (Nm or Nm/kg)	Comparison (uninjured limb or uninjured control group or preoperative rotation strength)
1	Seated	45	N/A	12	60/240	ER/IR, IR and ER	Nm/kg	Uninjured limb
2	Seated	0	10 mins	38	60/180/240	ER/IR, IR and ER	Nm/kg	Uninjured limb
3	Seated	45	6 mins	18	60/120/180	ER/IR, IR and ER	Nm	Uninjured limb, healthy control group, conservative group and preop
4	Seated	45	6 mins	18	60/120/180	IR and ER	Nm	Preop
5	Seated	0	N/A	38	60/180/240	ER/IR, IR and ER	Nm/kg	Uninjured limb
6	Seated	0	As much as needed	25	60/120/180	IR and ER	Nm	Uninjured limb
7	Seated	45	10 mins	15	30/60/240	ER/IR, IR and ER	Nm/kg	Uninjured limb, RTS status
8	Seated	90	N/A	15	60/180	IR and ER	N/A	Uninjured limb
9	Standing	0	N/A	4	60/180	IR and ER	Nm	Uninjured limb
10	Seated	45	6 mins	18	60/120/180	ER/IR, IR and ER	Nm/kg	Uninjured limb
11	Seated	0	N/A	N/A	30/90	IR and ER	N/A	Uninjured limb

Abbreviations: ER, external rotation; IR, internal rotation; N/A, not applicable.

#### Timing of post‐operative testing

The timing of isokinetic strength testing varied substantially. Two studies performed early postoperative testing at 4–5 months. Two studies performed testing at a single timepoint between 6 and 12 months after surgery, whereas four studies assessed isokinetic strength at follow‐up of ≥ 18 months. Three studies (Edouard 2010–2012) evaluated strength recovery cross‐sectionally with testing at 3, 6 and 21 months.

#### Rotational strength outcomes

Across the included studies, rotational strength recovery after the OL procedure demonstrated a temporal pattern.

In the early phase (< 6 months), all three studies (*n* = 102 patients) reported significant deficits in both ER and IR strength on the operated side. At 3 months, Edouard et al. observed the largest deficits, with ER reduced by 27%–30% and IR by 37%–42% across multiple testing speeds [8]. Moussa et al. reported similar but slightly smaller deficits at 4 months, with ER torque deficits of 8%–11% and IR torque deficits of 10%–14% [21]. Consistent with these findings, Reddy et al. demonstrated that at 5 months, only 30% of patients achieved acceptable limb symmetry (LSI < 10%) for both ER and IR at 60°/s and 180°/s, confirming that early postoperative rotational strength remains substantially impaired [24].

During the intermediate phase (6–12 months), all three studies reporting testing within this interval (*n* = 58 patients) demonstrated improvement in rotational strength compared with early postoperative testing, although residual deficits remained. Ribiero et al. reported significantly higher peak torque and maximum work for the uninjured shoulder at both 60°/s and 240°/s, indicating incomplete strength restoration at 6 months [25]. Caubère et al. similarly demonstrated persistent ER and IR deficits across 60°/s, 180°/s, and 240°/s [4]. Edouard et al. showed meaningful improvement between 3 and 6 months, although ER and IR torque remained significantly lower in the injured limb, even though fatigue indices normalised at this stage [8]. Together, these data indicate that while substantial recovery occurs during the intermediate phase, full symmetry is uncommon during the first postoperative year.

In the late phase (> 18 months), five studies reporting long‐term postoperative testing (*n* = 154 patients) reported small but measurable residual deficits, particularly in ER strength. Ernstbrunner et al. identified persistent peak torque deficits of 11% in ER and 4%–6% in IR across different testing speeds (60°/s, 180°/s and 240°/s) nearly 8 years after surgery, along with greater fatigability of the operated shoulder [10]. Malavolta et al. also found statistically significant ER deficits 38 months after surgery (13.4% at 60°/s; 4.5% at 180°/s), while IR strength had fully normalised [20]. These long‐term findings suggest that although IR strength recovers well over time, a small but persistent ER deficit might be a feature of long‐term strength recovery after the OL.

Across cohorts, rotational strength deficits were consistently larger at low angular velocities (30°/s–60°/s), while higher‐speed testing (180°/s–240°/s) showed earlier recovery and smaller side‐to‐side differences.

#### ER/IR Ratio

Eight studies reported the ER/IR ratio of the injured limb, with values ranging from 0.52 to 0.86. The ratio of the injured limb was elevated in studies that assessed early rotational strength recovery compared to the uninjured limb, reflecting a greater IR strength deficit, relative to the ER strength deficit at this stage. In studies that investigated late rotational strength recovery, the ER/IR ratio of the injured limb was slightly lower compared to the uninjured limb, reflecting residual ER weakness.

### Meta‐analysis

First, a pooled analysis was performed across all studies included in the meta‐analysis. When multiple postoperative time points were reported within a study, each time point was analysed separately rather than pooled. Next, subgroup analysis was performed for cohorts that underwent testing < 6 months (early‐phase group) and ≥ 12 months (late‐phase group) (Table 4).

**Table 4:** Meta‐analysis outcomes of isokinetic strength testing at 60°/s.

	External rotation strength		Internal rotation strength		ER/IR ratio			
	Mean ER torque deficit (%)	*p*‐Value	Mean IR torque deficit (%)	*p*‐Value	Injured limb	Uninjured limb	Mean difference (%)	*p*‐Value
Early‐phase	20.7	<0.01	18.3	<0.01	0.78	0.74	−6.14	0.012
Late‐phase	12.6	<0.01	9.1	<0.01	0.62	0.66	6.25	<0.01
Overall	17	<0.01	15.1	<0.01	0.69	0.7	1.61	0.25

Abbreviations: ER, external rotation; IR, internal rotation.

#### External rotation strength deficit

**Figure 2:**
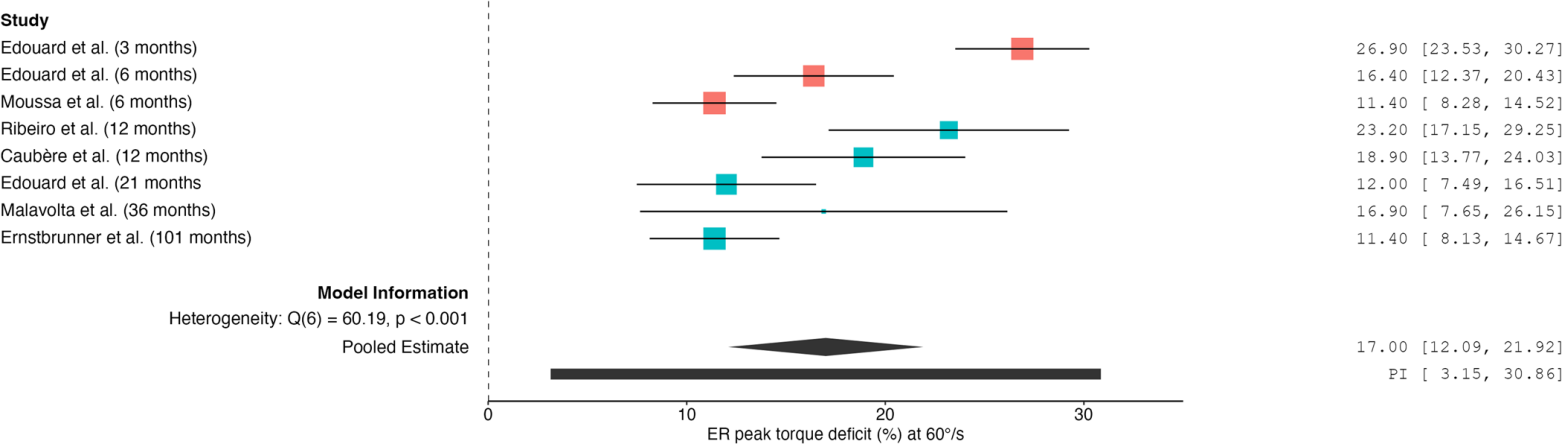
Forest‐plot of ER strength deficits (%), comparing early‐phase (red) and late‐phase (green) cohorts. ER, external rotation.

Eight independent cohorts (*n* = 252 shoulders) reported peak ER torque deficits at 60°/s [4, 8, 10, 20, 21, 25] (Figure 2).

The pooled mean ER deficit was 17.0% (95% CI, 12.1–21.9) with substantial between‐study heterogeneity (*I* ^2^ = 68.3%, *p* < 0.01).

Subgroup analysis revealed a clear temporal effect with a mean ER torque deficit in the early‐phase group (<6 months) of 20.7% (95% CI, 15.6–25.8) versus 12.6% (95% CI, 8.3–16.9) in the late‐phase group (≥12 months). This between‐group difference was statistically significant (*Qm* = 7.4, *p* = 0.006), indicating progressive recovery of ER strength over time. Despite this improvement, small side‐to‐side deficits persisted in late follow‐up cohorts.

#### Internal rotation strength deficit

**Figure 3:**
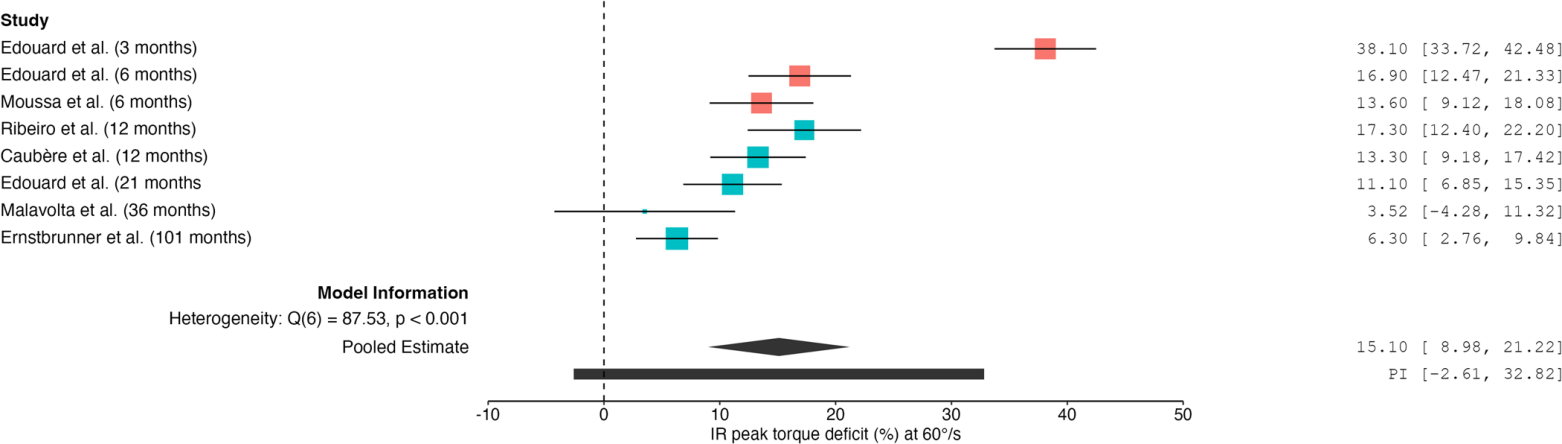
Forest‐plot IR strength deficits (%), comparing early‐phase (red) and late‐phase (green) cohorts. IR, internal rotation.

Across the same cohorts, the pooled mean IR deficit was 15.1% (95% CI, 9.0–21.2) with moderate heterogeneity (*I* ^2^ = 54.1%, *p* = 0.03) (Figure 3).

The early‐phase group showed a greater mean deficit of 18.3% (95% CI 12.0–24.6**)** compared to the late‐phase group with a mean deficit of 9.1% (95% CI 5.0–13.2, *Qm* = 6.2, *p* = 0.01). This pattern suggests partial but continuous recovery of subscapularis and synergistic IR strength over time, with near‐symmetrical strength levels achieved after 12 months.

#### ER/IR ratio differences

**Figure 4:**
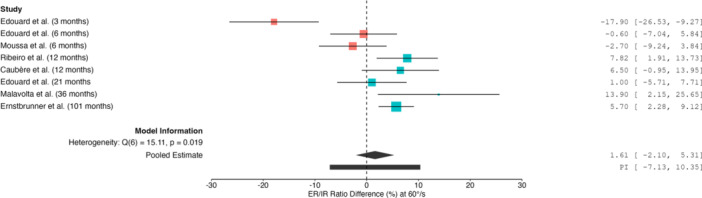
Forest‐plot ER/IR ratio differences (%), comparing early‐phase (red) and late‐phase (green) cohorts. ER/IR, external rotation/internal rotation.

Across all eight cohorts, the pooled mean ER/IR ratio of the injured limb was 0.69 ± 0.24 versus 0.72 ± 0.23 in the uninjured limb (Figure 4). This resulted in a pooled mean ER/IR ratio difference of 1.6% (95% CI, −2.1 to +5.3; *p* = 0.25) between the injured and uninjured limbs.

The overall ER/IR ratio demonstrated no significant asymmetry in global rotational balance. However, temporal subgroup analysis demonstrated a significant evolution of the ER/IR ratio over time (*p* = 0.002), confirming progressive normalisation of rotational balance beyond 6 months. In the early phase, the ER/IR ratio was slightly elevated compared to the uninjured limb, reflecting transient IR weakness. By the late phase, the ER/IR ratio had decreased to values below those of the uninjured limb, consistent with the small residual long‐term ER deficit. Together, these findings show that although absolute ER and IR torques remain asymmetric, their relative balance follows a predictable temporal pattern that ultimately returns toward physiological proportions.

Because only eight independent cohorts contributed to the quantitative synthesis, formal assessment of publication bias using funnel plots was not performed.

## DISCUSSION

The main finding of this study is that shoulder rotational strength recovery after the OL procedure follows a predictable temporal pattern. Early postoperative deficits are most pronounced in IR, resulting in a transient increase in the ER/IR ratio, whereas at longer follow‐up IR strength approaches symmetry and small but persistent external rotation deficits lead to a relative decrease in the ER/IR ratio. This reflects a characteristic shift in rotational balance over time.

The early IR strength deficit is most likely related to the split of the subscapularis muscle to expose the glenoid during surgery. Although this approach preserves tendon continuity and avoids the morbidity of a full tenotomy, it still disrupts muscle fibres and temporarily alters subscapularis tension and neuromuscular activation [1]. This mechanism is consistent with the more pronounced early IR deficits (up to 20%–28% compared with the contralateral side) observed across studies. Importantly, these deficits improve steadily, and most series show near‐complete normalisation of IR strength by 6–12 months. Current evidence therefore suggests that the subscapularis split produces a transient and reversible reduction in IR strength without any significant deficit at long‐term follow‐up.

On the other hand, ER strength demonstrates a different profile. Early postoperative ER deficits improve gradually but do not fully normalise, with several studies reporting residual deficits of 4%–15% at long‐term follow‐up. These small persistent asymmetries are not unique to OL and have also been reported after other stabilisation procedures such as Arthroscopic Bankart repair (ABR) [2, 17]. This suggests that a modest reduction in ER strength may represent a general feature of shoulder stabilisation surgery. In the context of the OL, several biomechanical factors—such as the sling effect of the conjoint tendon, subtle alterations in scapular resting position, and changes in glenohumeral translation—may also contribute to these persistent asymmetries, although their relative importance remains uncertain [3, 6, 11].

This temporal pattern has meaningful implications for rehabilitation. Since IR strength recovers predictably, rehabilitation should avoid over‐emphasising IR restoration. Instead, greater priority should be placed on the posterosuperior rotator cuff during the intermediate and late phases of rehabilitation. These muscles are essential for dynamic posterior stability and eccentric control during the deceleration phases of overhead and contact sports, where the posterior rotator cuff must absorb high distraction and shear loads while centring the humeral head in the glenoid [5, 22]. Given that a small but persistent ER deficit is the most consistent finding at long‐term follow‐up, insufficient posterior cuff strengthening may predispose athletes to overload, subtle kinematic adaptations, or increased instability risk despite good static stability. Late‐phase rehabilitation should therefore emphasise posterior cuff endurance, ER strength, and scapulothoracic control to align with the characteristic recovery profile identified in this review.

All included cohorts consisted of patients with recurrent anterior instability, typically with three or more dislocations before surgery. Recurrent anterior instability has been associated with rotator cuff muscle imbalance, alterations in neuromuscular activation, and deficits in shoulder proprioception [16, 18]. As a result, the postoperative strength deficits identified in this review likely reflect both surgical factors and pre‐existing alteration of neuromuscular control. Strength recovery patterns in recurrent‐instability cohorts should therefore not be extrapolated directly to first‐time dislocators, who generally demonstrate less preoperative strength deficits and faster neuromuscular recovery after stabilisation procedures.

There was considerable heterogeneity in surgical technique, rehabilitation, and testing protocols (angular velocities (30°/s–240°/s), arm positions, and torque reporting methods) highlighting the need for standardisation to improve comparability and enable robust pooled analyses. Nevertheless, the direction and magnitude of findings were consistent across studies. High‐speed testing (≥180°/s) typically revealed smaller limb asymmetries, suggesting earlier recovery of dynamic neuromuscular coordination relative to maximal torque generation.

This review has several limitations. The number of unique cohorts remains modest, and no randomised comparisons between the OL procedure and ABR are available. Preoperative strength status, surgical indications, and rehabilitation adherence were inconsistently reported, limiting adjustment for confounders. The study populations predominantly consisted of male contact athletes with recurrent instability, reducing generalisability to female patients, first‐time dislocators or recreational non‐contact populations. The exact location of the subscapularis split (e.g., middle versus inferior third) was inconsistently reported and could therefore not be analysed, although this may represent another source of variability influencing internal rotation recovery. Overall, the certainty of evidence across outcomes is limited by the predominance of observational study designs, moderate risk of bias, and methodological heterogeneity among the included studies. Finally, protocol heterogeneity precluded full quantitative pooling, although structured meta‐analytic synthesis was feasible for key outcomes.

Future studies should standardise isokinetic protocols, directly compare open and arthroscopic techniques, and include more diverse patient populations. Longitudinal analyses with serial testing from 3 months to ≥12 months are required to define recovery trajectories more precisely and to determine whether persistent ER deficits have meaningful implications for long‐term shoulder function and stability.

## CONCLUSION

Shoulder rotational strength recovery after the OL procedure through a subscapularis‐split approach follows a predictable temporal pattern, with early internal rotation deficits resolving by 6–12 months and small but persistent external rotation deficits at long‐term follow‐up. These findings indicate that the split of the subscapularis muscle does not compromise IR strength at long‐term follow‐up, supporting its safety for young and active patients.

## AUTHOR CONTRIBUTIONS

**Willem Geuskens**: Conceived the study, coordinated the systematic review process, performed data analysis, drafted the manuscript, conducted the literature search, screened studies for eligibility and performed quality assessment. **Arjun Mehra**: Conducted the literature search, screened studies for eligibility and performed quality assessment. **Eoghan Hurley**: Contributed substantially to the study design and interpretation of the results and provided critical revision of the manuscript. **Hannan Mullett**: Contributed substantially to the study design and interpretation of the results and provided critical revision of the manuscript.

## CONFLICT OF INTEREST STATEMENT

The authors declare no conflicts of interest.

## ETHICS STATEMENT

The authors have nothing to report. PROSPERO registration number: CRD420251248278.

## Supporting information

Table A1: Database‐specific search strategies used for the systematic review.

## Data Availability

Data sharing is not applicable to this article as no new data were generated or analysed in this study. All data underlying the findings are derived from previously published studies and are available within the article and its supplementary materials.
