# Supplementary material for: Internal rotation strength recovers fully after the open Latarjet via a subscapularis‐split: A systematic review and meta‐analysis
Source: J Exp Orthop. 2026 Aug 6;13(3):e70882. doi: 10.1002/jeo2.70882 (PMC13446457; doi:10.1002/jeo2.70882)
Supplement: Supplementary file 1 [file JEO2-13-e70882-s001.docx]

| **Database** | **Search Query** |
| --- | --- |
| PubMed | 1. ((Latarjet) AND (Isokinetic strength)) AND (("1900/01/01"[Date - Publication] : "2026/04/01"[Date - Publication])) 2. ((Latarjet) AND (shoulder rotation strength)) AND (("1900/01/01"[Date - Publication] : "2026/04/01"[Date - Publication])) |
| Cochrane | 1. latarjet in Title Abstract Keyword AND shoulder rotation strength in Title Abstract Keyword - with Cochrane Library publication date Between Jan 1900 and Apr 2026 2. latarjet in Title Abstract Keyword AND isokinetic strength in Title Abstract Keyword - with Cochrane Library publication date Between Jan 1900 and Apr 2026 |
| Embase | 1. latarjet AND ('isokinetic strength'/exp OR 'isokinetic strength' OR (isokinetic AND ('strength'/exp OR strength))) AND [01-01-1983]/sd NOT [02-04-2026]/sd 2. latarjet AND ('shoulder rotation strength' OR (('shoulder'/exp OR shoulder) AND ('rotation'/exp OR rotation) AND ('strength'/exp OR strength))) AND [01-01-1983]/sd NOT [02-04-2026]/sd |
